# Tailoring Solvation Sheaths and Interfacial Chemistry: A Review of Electrolyte Engineering for Highly Reversible Aqueous Zinc–Iodine Batteries

**DOI:** 10.3390/molecules31122127

**Published:** 2026-06-17

**Authors:** Huayang Zhou, Tianhao Yu, Shaojie Zhang, Zhou Jiang, Kaiming Zhou, Zizhen Liu, Qiaoya Han, Yanjun Wen, Yang Wang

**Affiliations:** 1 Intelligent Manufacturing of Functional Chemicals Engineering Research Centre of the Ministry of Education, College of Chemistry, Chemical Engineering and Materials Science, Shandong Normal University, Jinan 250014, China; 2 Institute of Catalysis Research and Technology, Karlsruhe Institute of Technology (KIT), Hermann-von-Helmholtz-Platz 1, 76344 Eggenstein-Leopoldshafen, Germany

**Keywords:** aqueous zinc–iodine batteries, electrolyte engineering, solvation structure, polyiodide shuttle effect, interfacial chemistry, multi-electron conversion, in situ characterization

## Abstract

Aqueous zinc–iodine batteries (AZIBs) are emerging as highly promising candidates for next-generation, grid-scale energy storage due to the intrinsic safety of water-based electrolytes, the high theoretical capacity of the zinc anode, and the rapid conversion kinetics of the iodine cathode. However, the practical commercialization of AZIBs is severely impeded by formidable interfacial instabilities, including the uncontrollable growth of zinc dendrites, parasitic hydrogen evolution reactions (HER), and the notorious polyiodide (I_3_^−^, I_5_^−^) shuttle effect. These macroscopic degradation modes are fundamentally rooted in the robust [Zn(H_2_O)_6_]^2+^ primary solvation sheath and the immense thermodynamic driving force for polyiodide dissolution in highly polar aqueous media. To address these interconnected challenges, electrolyte engineering has evolved into the most potent, holistic strategy. This comprehensive review systematically evaluates the latest advancements in electrolyte engineering for AZIBs. We first deeply decipher the fundamental thermodynamic mechanisms governing Zn^2+^ desolvation and iodine multiphase conversion. Subsequently, we critically analyze cutting-edge regulation paradigms, including water-in-salt (WIS) and localized high-concentration electrolytes (LHCE), cosolvent networks, functional molecular additives, deep eutectic solvents (DES), and quasi-solid-state hydrogels. By integrating in situ/operando spectroscopic characterizations with multiscale theoretical computations (such as MD and DFT), we elucidate the structure–activity relationships at the atomic level. Finally, we provide strategic perspectives on the future trajectories of the field, emphasizing the stabilization of multi-electron (I^−^/I^0^/I^+^) halogen chemistry, AI-driven high-throughput screening, and the rigorous standardization of Ah-level pouch cell engineering for extreme-environment applications.

## 1. Introduction

Industrial growth and changes in global energy create environmental issues. Thus, we urgently need reliable energy storage for the power grid [1,2]. Lithium-ion batteries (LIBs) are widely used in electronics and cars. However, their use in large energy storage is limited by flammable organic electrolytes, high costs, and limited lithium resources. Therefore, water-based batteries are a good alternative. They use safe water electrolytes, which prevents fire risks [3]. Among many water-based metal-ion batteries (like Na^+^, K^+^, Mg^2+^, and Al^3+^), aqueous zinc batteries are very attractive because of the zinc anode. Zinc has a high specific capacity (820 mAh g^−1^, 5854 mAh cm^−3^) and a low redox potential (0.76 V vs. standard hydrogen electrode, SHE). It is also stable and easy to source globally [4].

Aqueous zinc–iodine batteries (AZIBs) receive a lot of attention today. Common cathodes, like vanadium or manganese oxides, break down easily and have slow ion movement. The iodine cathode is different. It uses a fast and reversible solid–liquid–solid change (3I^−^ ⇌ I_3_^−^ + 2e^−^; 2I_3_^−^ ⇌ 3I_2_ + 2e^−^). This chemistry gives AZIBs fast reactions, flat voltage curves, and long cycle life. These features make them good for high-power energy storage [5,6].

But AZIBs still face problems before they can be sold. They have unstable interfaces and break down easily during use. These issues come from how the active materials react with the water electrolyte [7]. On the cathode side, solid iodine (I_2_) and iodide (I^−^) change into polyiodides, mainly triiodide (I_3_^−^) and pentaiodide (I_5_^−^). Because they are highly polar, these polyiodides dissolve easily in water. The dissolved I_3_^−^ ions move away from the cathode and pass through the separator due to differences in concentration and the electric field [8]. This is called the “polyiodide shuttle effect.” It causes a permanent loss of cathode materials and lowers the Coulombic efficiency (CE) [9].

At the same time, the anode faces different problems because free water molecules (H_2_O) are very active. In standard water electrolytes (like 1 M or 2 M ZnSO_4_), the Zn^2+^ ion binds tightly with six water molecules. This forms a strong shell known as [Zn(H_2_O)_6_] ^2+^. When zinc deposits, it takes a lot of energy to remove these water molecules. Also, the water in this shell breaks down easily. This causes a side reaction where hydrogen gas forms (HER). It happens near the voltage where zinc plates [10]. The HER reaction uses up protons, which makes the local area more basic (higher pH). This causes solid, useless byproducts like zinc hydroxide sulfate to form. Also, zinc ions do not deposit evenly. They gather on small bumps, making sharp zinc dendrites grow fast. These sharp dendrites can poke through the separator. This causes short circuits and ruins the battery [11].

Recent studies show that AZIB failure is not just one problem at one electrode. Instead, the problems at both electrodes affect each other. This is called “electrode–electrolyte crosstalk” [12,13]. When dissolved I_3_^−^ moves to the anode, it reacts directly with the zinc metal (Zn + I_3_^−^ → Zn^2+^ + 3I^−^). This creates a chemical short circuit. It eats away the new zinc and uses up the active iodine. The battery loses charge on its own very quickly, which makes it hard to use for large storage [14].

In the past, researchers tried to fix these issues using physical blocks. For the cathode, they put iodine inside porous materials like carbon, MXenes, or MOFs. This traps the polyiodides using small spaces or weak bonds. For the anode, they built artificial protective layers (SEI) to keep water away from the zinc metal. But these physical fixes do not solve the main problem. The materials still want to dissolve, and water still wants to break down. Also, adding a lot of inactive carbon makes the battery larger and lowers its total energy [15].

Because of this, changing the electrolyte itself has become a major new approach for AZIBs. Instead of just using the electrolyte to move ions, new research changes its atomic structure and physical traits. This helps control how the battery reacts [1]. Researchers use new designs like water-in-salt (WIS), localized high-concentration electrolytes (LHCE), deep eutectic solvents (DES), or special additives. This allows them to solve multiple problems at once. First, some electrolyte parts can hold onto the iodine. This changes how iodine dissolves and stops the shuttle effect. Second, replacing water in the Zn^2+^ shell with other ions or molecules makes the water less active. It reduces gas formation and helps zinc deposit smoothly without dendrites [12].

Also, these new electrolytes are pushing the limits of what AZIBs can do. Normal AZIBs use a two-electron process (I^−^/I_2_). But new findings show that changing the electrolyte can protect the active iodine cation (I^+^). By adding strong binding agents, the I^+^ does not react badly with water. This enables a stable four-electron process (2I^−^/I_2_/2I^+^). This big change greatly increases the capacity and total energy of AZIBs [16,17].

Given these quick advances, this review aims to clearly summarize the progress in AZIB electrolyte design. First, we will look at the basic chemistry of how Zn^2+^ dissolves and how iodine behaves. Next, we will review current ways to improve electrolytes, like using high-concentration salts, additives, and solid-like gels. Finally, we discuss the tools and computer models used to understand these changes. This provides a guide for making high-energy, long-lasting AZIBs for the market in the future.

## 2. Fundamental Mechanisms

To design stable electrolytes for AZIBs, we must first understand how the active materials behave in water. AZIBs fail in several ways. Zinc dendrites grow fast. The battery loses charge because polyiodides move around. Side reactions also use up the electrolyte. These are not simple design errors. They happen because of chemical bonds, the way ions dissolve, and how molecules interact in the fluid and at the electrodes [18,19].

### 2.1. The Primary Solvation Sheath of Zn^2+^ and Hydrogen-Bonding Networks

In standard weak water electrolytes (like 1.0 M or 2.0 M ZnSO_4_ or Zn(CF_3_SO_3_)_2_), water molecules control the chemical setting. Water has high polarity and a large dielectric constant (ε ≈ 78 at room temperature). It also shares electrons easily, making it a very active solvent. Strong electrical forces act between the Zn^2+^ ions and the polar H_2_O molecules. This interaction forms the main structure of the fluid [20,21]. Beyond the localized constraints of the primary solvation sheath, the macroscopic electrochemical behavior of the electrolyte is heavily dictated by the bulk fluid dynamics. The hydrogen atoms of the coordinated water connect with the oxygen atoms of nearby free water molecules, generating an extensive, highly dynamic 3D hydrogen-bonding network across the entire electrolyte. This ubiquitous network plays three critical roles in limiting battery performance. First, it acts as a highly efficient “fast track” for rapid proton transport via the Grotthuss mechanism, which kinetically accelerates the parasitic Hydrogen Evolution Reaction (HER) at the anode interface. Second, this continuous networking drastically amplifies the chemical activity of the free water molecules, fundamentally restricting the thermodynamic electrochemical stability window of the electrolyte and limiting the stable battery voltage to a narrow 1.23 V. Finally, on the cathode side, this highly polar, interconnected water environment provides the immense thermodynamic driving force required to solvate highly polar polyiodide species (I^3−^, I^5−^). The intense dipole interactions from the bulk water forcefully pull these active iodine species out of the cathode matrix, directly initiating the catastrophic polyiodide shuttle effect.

Computer models, like Density Functional Theory (DFT) and Molecular Dynamics (MD), show clear details of this state at the atomic level. In a normal weak liquid, one Zn^2+^ ion binds tightly with six water molecules. The oxygen atoms in water share their electrons with the empty spaces in the Zn^2+^ ion. This shape makes a very stable and rigid shell. It is called [Zn(H_2_O)_6_]^2+^ [22]. The strong [Zn(H_2_O)_6_]^2+^ shell fundamentally dictates both the stability of the electrolyte and the kinetics of ion transport. From a stability perspective, the intense electric field at the electrode heavily distorts the electron clouds of the coordinated water molecules, weakening their O-H bonds. Consequently, these bound water molecules are far more susceptible to reductive decomposition than bulk free water, triggering parasitic hydrogen evolution reactions (HER) prior to actual zinc plating. This localized water consumption rapidly elevates the interfacial pH, driving the formation of insulating byproducts like zinc hydroxide sulfate that passivate the anode. From an ion transport perspective, the massive binding energy of the [Zn(H_2_O)_6_]^2+^ complex creates a formidable thermodynamic desolvation barrier. This not only results in sluggish charge transfer kinetics but also contributes to uneven spatial ion flux. Driven by the “tip-effect,” the sluggishly desolvating ions preferentially accumulate at high-energy surface protrusions, catastrophically accelerating the growth of sharp zinc dendrites.

The bond energy in this shell is very high, often over −400 kJ mol^−1^. This makes the shell hard to break at room temperature. Also, this structure affects the fluid around it. The water molecules in the [Zn(H_2_O)_6_]^2+^ shell are not alone. Their hydrogen atoms connect with oxygen atoms from nearby free water molecules. This creates a large, changing 3D network of hydrogen bonds across the whole fluid [23]. It is a common electrochemical paradox that conventional dilute aqueous electrolytes exhibit a narrow thermodynamic stability window of approximately 1.23 V not despite, but rather because of, water’s high dielectric constant ≈ 78) and intense polarity. While these properties make water an exceptional solvent for salt dissociation, they also render H_2_O molecules extremely sensitive to interfacial electric fields. When polar water molecules approach the highly charged electrode interface, the intense local electric field severely distorts their electron clouds, further polarizing and fundamentally weakening the intramolecular O-H bonds. This distortion significantly lowers the activation energy required for bond cleavage, causing water to undergo reductive hydrogen evolution (HER) and oxidative oxygen evolution (OER) at remarkably low overpotentials. Furthermore, the high dielectric constant ensures an overwhelming abundance of free water, which sustains the dynamic, long-range hydrogen-bonding network that kinetically accelerates these parasitic decomposition reactions via rapid proton hopping. Consequently, the restricted 1.23 V window is an unavoidable thermodynamic consequence of water’s intrinsic polar and dielectric nature.

This large network acts like a fast track for moving protons. It also makes the free water molecules much more active [24,25]. This connected and active water environment is the main reason why normal water electrolytes have a narrow working range. It limits the stable battery voltage to just 1.23 V.

### 2.2. Thermodynamics and Interfacial Kinetics of Zn^2+^ Desolvation

The strong [Zn(H_2_O)_6_]^2+^ shell makes zinc plating difficult. For a zinc ion to plate on the anode, it goes through several steps. First, the [Zn(H_2_O)_6_]^2+^ complex moves from the main fluid to the edge of the electrode. When it reaches the negative surface, it must drop its bound water molecules. It becomes a bare Zn^2+^ ion and then takes two electrons to join the solid metal [26,27].

Dropping the water molecules is called desolvation. This step needs a lot of energy. It is hard to pull the polar H_2_O molecules away from the Zn^2+^ ion. At the electrode, the strong electric field twists the electron clouds of the bound water molecules. This twisting weakens the O-H bonds inside the water. As a result, these bound water molecules break down much easier than the free water in the main fluid [28].

Because of this, the bound water breaks down before the zinc ion can plate cleanly. This local breakdown causes a bad side reaction called hydrogen evolution (HER). The reaction is: 2H_2_O + 2e^−^ → H_2_ ↑ + 2OH^−^. The voltage for plating zinc (0.76 V vs. SHE) is lower than the voltage for making hydrogen gas. So, HER happens easily in slightly acidic water [29,30].

**Figure 1:**
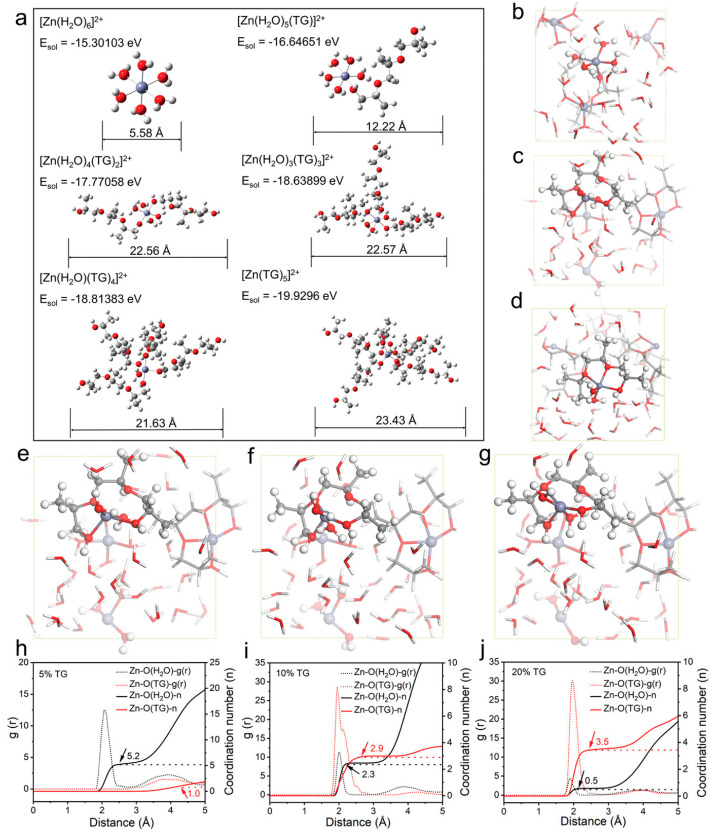
The theoretical calculation of solvation energy and AIMD simulation were performed to verify the involvement of TG in the solvation sheath structure. (**a**) Dimensions and solvation energies of Zn^2+^ with different coordination structures. Snapshots of the AIMD simulation for (**b**) 5% TG, (**c**) 10% TG, and (**d**) 20% TG electrolytes, showing the interaction of TG with Zn^2+^. Snapshots of the solvation sheath and surrounding chemical environment in 10% TG electrolyte at (**e**) 30.0, (**f**) 40.0, and (**g**) 50.0 ps, respectively. Radial distribution functions and coordination numbers of Zn-O (TG) and Zn-O (H_2_O) in the (**h**) 5% TG, (**i**) 10% TG, and (**j**) 20% TG electrolytes. Element colors: dark blue for Zn, gray for C, white for H, red for O [31].

The HER reaction uses up protons (H^+^). This makes the local area quickly become basic with a higher pH. When zinc salts like ZnSO_4_ are present, this basic setting creates solid byproducts. These byproducts do not dissolve. The most common one is zinc hydroxide sulfate (e.g., Zn_4_SO_4_ (OH)_6_·xH_2_O) [31]. These solid layers cover the anode surface. They block the active spots and stop new Zn^2+^ ions from reaching the metal evenly (Figure 1).

This uneven flow of ions causes a big problem. Zinc atoms prefer to move to high-energy edges on the surface. This causes the “tip-effect.” The electric field becomes stronger at tiny bumps on the metal. This makes zinc plate there even faster. Soon, sharp zinc dendrites grow out of control [32]. To fix this and achieve stable zinc plating, we must change the primary solvation shell. We can do this by adding organic molecules or using many specific anions. These additives push the H_2_O molecules away from the central zinc ion.

A critical evaluation of electrolyte engineering must rigorously distinguish between the absolute transport kinetics (ionic conductivity, σ, and absolute diffusion, D) and the relative charge-carrying fraction (transference number, t_Zn2+_). In conventional dilute aqueous electrolytes, Zn^2+^ is typically low (approximately 0.3–0.4). This occurs because the highly hydrated [Zn(H_2_O)_6_]^2+^ complex possesses a massive hydrodynamic radius, resulting in lower ionic mobility (μ_Zn_) compared to the smaller, less-hydrated counter-anions (μ_anion_).

Many advanced electrolyte strategies—such as Water-in-Salt (WIS), Deep Eutectic Solvents (DES), and densely cross-linked hydrogels—frequently report significantly enhanced Zn^2+^ values (often exceeding 0.7). However, the physical origin of this enhancement does not imply that Zn^2+^ moves “faster” in absolute terms. Rather, these heavily engineered microenvironments introduce massive ionic aggregates, strong electrostatic anchoring, or tortuous steric channels that selectively exert disproportionate friction on the counter-anions. By drastically severely restricting anion mobility (μ_anion_), the mathematical ratio of the current carried by the cations (t_Zn2+_) = μ_Zn_/(μ_Zn_ + μ_anion_) inherently increases.

This introduces a profound stability transport paradox. According to the Stokes–Einstein relationship and Walden’s rule, the drastic increase in macroscopic viscosity (η) engineered to suppress free water activity and interfacial corrosion simultaneously inflicts a severe kinetic penalty. The intense viscosity dramatically depresses the absolute diffusion coefficients (D_Zn_) of all ionic species, leading to a precipitous drop in bulk ionic conductivity (σ). Therefore, while a high Zn^2+^ is fundamentally highly beneficial for mitigating localized concentration polarization and delaying the onset of dendrite nucleation according to Sand’s time model, the severely sluggish absolute transport in these viscous systems inherently restricts the high-rate, high-current-density operation of practical AZIBs. Recognizing and decoupling this viscosity–conductivity trade-off—such as through the deployment of Localized High-Concentration Electrolytes (LHCE) or specifically charged porous membranes—remains one of the most formidable challenges in the field.

### 2.3. Multiphase Conversion Thermodynamics and Shuttling Mechanisms of Iodine

The anode has surface problems, but the cathode also faces hard chemistry issues with iodine. Changing solid iodine (I_2_) to iodide (I^−^) is not a simple one-step reaction. Instead, it goes through a “solid–liquid–solid” process. During discharge, solid I_2_ changes into polyiodide ions. These are mainly triiodide (I_3_^−^) and pentaiodide (I_5_^−^). These middle steps dissolve very easily in water. The reactions are: 3I^−^ ⇌ I_3_^−^ + 2e^−^ and 2I_3_^−^ ⇌ 3I_2_ + 2e^−^ [33].

The main problem for AZIBs is that these polyiodides dissolve too well in water. In pure water, the energy change for dissolving I_3_^−^ and I_5_^−^ is very large and negative. The water molecules pull strongly on the polyiodide chains. This force makes the iodine species leave the tiny holes of the carbon cathode [34,35].

Once they dissolve into the main fluid, two forces push these active species. First, there is much more iodine near the cathode than in the rest of the fluid. This difference pushes the I_3_^−^ ions outward through normal spreading. Second, during discharge, the electric field pulls the negative polyiodides toward the anode side [36].

This movement is called the polyiodide shuttle effect. It drains the battery from the inside. The cathode loses its active materials permanently. The battery also loses charge quickly when resting [33,37]. Also, the reactions between I_2_ and I_3_^−^ can become very slow in real-world use. This happens when the battery has a thick iodine layer or runs at high currents. Because the reaction is slow, polyiodides build up near the surface. This buildup makes them dissolve outward even faster [34].

**Figure 2:**
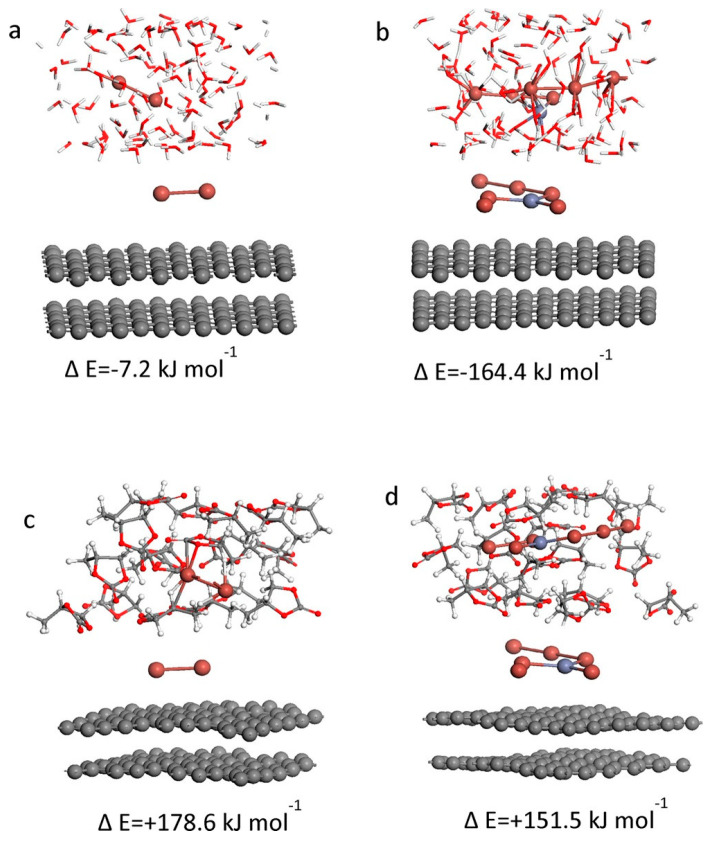
DFT calculations of the interaction energy difference (Δ*E*) between adsorption on the carbon surface and dissolution in different solvents. I_2_ and Zn(I_3_)_2_ in (**a**,**b**) water and (**c**,**d**) PC solvent, respectively [16].

Therefore, just putting iodine inside porous carbon is not enough to stop this process. We must change the chemistry of the electrolyte so it does not dissolve the iodine species so easily. In general, the adsorption of iodine species onto the carbon surface and their dissolution in water solvents compete with each other. In the DFT simulation, the interaction energy difference (Δ*E*) of each species in the above two processes (adsorption and solvation) was calculated (Figure 2a,b). Iodine species (i.e., I_2_ and Zn(I_3_)_2_) show negative Δ*E* values of −7.2 and −164.4 kJ mol^−1^ in water, respectively, indicating that they are preferentially adsorbed onto carbon rather than dissolved in water solvents due to their stronger interaction with the carbon surface [16]. On the contrary, I_2_ and Zn(I_3_)_2_ exhibit significantly positive Δ*E* values in an organic solvent such as propylene carbonate (PC), suggesting that they are more likely to be dissolved in solvent rather than to interact with the carbon surface due to the much stronger solvating interaction in the PC solvent (Figure 2c,d) [16].

To understand the true limits of aqueous zinc–iodine chemistry, the focus must shift from generic anode passivation to highly specialized cathode–electrolyte interactions (CEI) and polyiodide speciation. Traditional iodine battery configurations rely almost exclusively on the physical confinement of solid iodine (I_2_) within porous carbon matrix hosts to mitigate the shuttle effect. However, physical adsorption alone cannot overcome the immense thermodynamic driving force for polyiodide dissolution in highly polar aqueous media. Electrolyte engineering fundamentally redefines this boundary through thermodynamic and chemical confinement. For instance, in Water-in-Salt (WIS) and Localized High-Concentration Electrolytes (LHCE), the radical elimination of bulk free water creates an “anti-solvent” effect. Because highly polar intermediates like triiodide (I_3_^−^) and pentaiodide (I_5_^−^) are strictly starved of their mandatory hydration sheaths, the dissolution equilibrium is forced backward, chemically pinning the active materials within the porous cathode architecture. Simultaneously, specific dual-function molecular additives and cosolvents dynamically interact with the cathode host to form a protective, ionically conducting Cathode–Electrolyte Interphase (CEI). This in situ generated CEI alters the local dielectric properties at the triple-phase boundary, screening the active iodine species from nucleophilic attack by residual water molecules and suppressing detrimental self-discharge.

Furthermore, it is an oversimplification to assume that iodine speciation is strictly limited to I_3_^−^ and I_5_^−^. In highly concentrated or heavily engineered electrolyte environments—particularly those utilizing mixed-halide salts (e.g., containing ZnCl_2_ or ZnBr_2_) or operating under high anodic voltages—the halogen chemistry becomes exceptionally complex. In these regimes, the active iodine species readily interact with foreign halide anions to form mixed interhalogen complexes, such as ICl_2_^−^, IBr_2_^−^, or even higher-order polyhalides. The formation of these interhalogens fundamentally alters the standard multiphase conversion thermodynamics, shifting the equilibrium redox potentials and modifying the solubility profiles of the active species. Under intense electric fields or slightly alkaline localized pH, various oxyiodine species (e.g., IO^−^) may transiently form, further complicating the interfacial chemistry and necessitating advanced in situ spectroscopic techniques to precisely map the reaction pathways. Successful four-electron operation strictly requires specialized electrolyte design—such as the introduction of strong Lewis-basic complexing agents or quaternary ammonium compounds—to encapsulate the emerging I^+^ species within a rigid coordination pocket, lowering its lowest unoccupied molecular orbital (LUMO) energy level and completely suppressing the hydrolysis pathway to ensure highly reversible multielectron halogen electrocatalysis.

### 2.4. Crosstalk Mechanisms: The Intersection of Anode and Cathode Failures

Scientists now agree that the failures at the zinc anode and the iodine cathode are linked. This overall damage is caused by “electrode–electrolyte crosstalk” [19,33]. When the dissolved I_3_^−^ ions pass through the separator and reach the anode, they are very reactive. They quickly react with the zinc metal (Zn + I_3_^−^ → Zn^2+^ + 3I^−^).

This cycle creates a constant chemical short circuit inside the battery. It eats away the fresh zinc metal and turns it into dissolved Zn^2+^ ions. At the same time, it changes the polyiodides back into I^−^. This reaction gives no power to the outside device. This harmful crosstalk also changes the local fluid chemistry. It alters the salt levels and pH. These changes then speed up gas formation and dendrite growth even more.

Thus, a good electrolyte design must treat the whole system. It needs to change the Zn^2+^ shell to stop water breakdown at the anode. At the same time, it must lower the fluid’s holding power or change its physical traits to trap the polyiodides at the cathode.

## 3. Electrolyte Engineering Strategies

Having established the fundamental degradation mechanisms dictated by the aqueous thermodynamic environment—specifically the robust [Zn(H_2_O)_6_]^2+^ primary solvation sheath and the immense thermodynamic driving force for polyiodide dissolution—it becomes unequivocally clear that passive, localized physical protections at the electrode level are insufficient to propel AZIBs toward practical commercialization. Consequently, electrolyte engineering has rapidly evolved into the most potent, proactive, and holistic strategy to simultaneously stabilize the Zn anode and entirely arrest the polyiodide shuttle effect. By fundamentally manipulating the coordination chemistry, altering the macroscopic physical properties (such as dielectric constant and donor number), and redesigning the thermodynamic equilibrium of the electrolyte, researchers can achieve comprehensive interfacial regulation. This extensive section critically evaluates the five most prominent and cutting-edge electrolyte engineering paradigms in the current development of highly reversible AZIBs, providing a deep dive into their molecular-level mechanisms and macroscopic electrochemical consequences.

### 3.1. High-Concentration and Localized High-Concentration Electrolytes (WIS & LHCE)

The conceptualization of “Water-in-Salt” (WIS) electrolytes, initially pioneered to expand the electrochemical stability window of aqueous lithium-ion batteries, has been successfully and transformatively transplanted to zinc-based systems to suppress parasitic water activity [38,39]. In a typical dilute aqueous electrolyte, such as 1.0 M or 2.0 M ZnSO_4_, the molar ratio of solvent H_2_O molecules to solute Zn^2+^ ions is overwhelmingly high (frequently exceeding 50:1). This abundance of free water leads to uncontrollable hydrogen evolution reactions (HER) and active dissolution of iodine species. However, in a WIS electrolyte, this ratio is drastically reduced, completely overturning the traditional solvent-in-salt paradigm. Simultaneously, for the iodine cathode, the WIS strategy provides a phenomenal thermodynamic advantage. In a standard dilute electrolyte, highly polar polyiodide intermediates (I_3_^−^, I_5_^−^) rely heavily on strong dipole–ion interactions with abundant free water molecules to dissolve and migrate. However, in a WIS system, the near-total elimination of free water drastically reduces the macroscopic dielectric constant of the bulk solvent. This severe lack of free solvent molecules fundamentally shifts the thermodynamic equilibrium of the multiphase conversion reaction. The active iodine species are effectively “starved” of solvation sites. Consequently, the heavily concentrated electrolyte functions as an “anti-solvent” for polyiodides. This immense thermodynamic barrier forces the polyiodides to rapidly precipitate back onto the porous carbon host matrix rather than dissolving into the bulk fluid, thereby physically and thermodynamically halting the notorious shuttle effect at its source.

By dissolving exceptionally high concentrations of bulky, highly soluble, and low-lattice-energy salts—most notably fluorinated salts such as zinc bis(trifluoromethanesulfonyl)imide, Zn(TFSI)_2_, or zinc trifluoromethanesulfonate, Zn(OTf)^2−—^the inherent solvation structure undergoes a radical, systemic transformation [40,41]. At super-high salt concentrations (e.g., 1 M Zn(TFSI)_2_ mixed with 20 M LiTFSI to create a massive ionic strength), there is a severe deficit of free water molecules to fully hydrate all the cations present in the system. Consequently, the bulky fluorinated anions (TFSI^−^ or OTf^−^) are thermodynamically forced to penetrate the primary solvation sheath of the Zn^2+^ cations. They directly displace the coordinated H_2_O molecules to form robust contact ion pairs (CIPs) and intricate cation–anion aggregates (AGGs) [42]. Furthermore, during the initial electrodeposition cycles, the altered coordination environment fundamentally shifts the interfacial reduction thermodynamics. Because the bulky fluorinated anions (TFSI^−^ or OTf^−^) are forced into the primary solvation sheath, their lowest unoccupied molecular orbital (LUMO) energy levels are lowered, rendering them more susceptible to electrochemical reduction than the shielded water molecules. Consequently, these coordinated fluorinated anions are preferentially reduced at the anode/electrolyte interface. This targeted decomposition triggers the in situ formation of a highly dense, inorganic-rich Solid Electrolyte Interphase (SEI) predominantly composed of zinc fluoride (ZnF_2_) and zinc sulfide (ZnS), embedded within protective fluorinated polymeric species. This anion-derived SEI provides unparalleled interfacial stability: it is strictly electronically insulating, which permanently halts parasitic electron transfer to bulk water and suppresses HER, while remaining highly ionically conductive to facilitate rapid Zn^2+^ transport. Ultimately, this robust interphase entirely isolates the fresh zinc metal from the corrosive bulk aqueous environment and crystallographically guides highly reversible, epitaxial Zn^2+^ plating parallel to the low-surface-energy (002) plane, physically and thermodynamically suppressing dendrite propagation.

This profound anion-rich coordination environment significantly decreases the highest occupied molecular orbital (HOMO) energy of the remaining coordinated water molecules. From a spectroscopic perspective, Raman and Fourier-transform infrared (FTIR) spectroscopy consistently reveal a dramatic blue-shift and narrowing of the O-H stretching vibration bands, indicating the severe disruption of the ubiquitous three-dimensional hydrogen-bonding network that typically facilitates rapid proton transport via the Grotthuss mechanism [43]. For the zinc anode, this means that the desolvation energy barrier is fundamentally altered.

Furthermore, during the initial electrodeposition cycles, the coordinated fluorinated anions (TFSI^−^ or OTf^−^) are preferentially reduced at the anode/electrolyte interface before water molecules. This triggers the in situ formation of a dense, inorganic-rich Solid Electrolyte Interphase (SEI) predominantly composed of ZnF_2_, ZnS, and protective fluorinated polymeric species. This robust, ionically conductive, and electronically insulating SEI entirely isolates the fresh zinc metal from the bulk aqueous environment, guiding highly reversible, epitaxial Zn^2+^ plating parallel to the (002) crystallographic plane and physically suppressing dendrite propagation [44,45].

Simultaneously, for the iodine cathode, the WIS strategy provides a phenomenal thermodynamic advantage. The near-total elimination of free water drastically reduces the macroscopic dielectric constant of the bulk solvent. This severe lack of free solvent molecules shifts the thermodynamic equilibrium of the multiphase conversion reaction. Polyiodide species (such as I_3_^−^ and I_5_^−^), which inherently rely on strong dipole–ion interactions with water to dissolve, are essentially “starved” of solvation sites. The electrolyte essentially acts as an anti-solvent for polyiodides, forcing them to rapidly precipitate back onto the porous carbon host matrix rather than dissolving into the bulk fluid, thus physically and thermodynamically halting the notorious shuttle effect [46].

Despite these remarkable scientific achievements, the practical, large-scale commercialization of WIS electrolytes in AZIBs is severely hampered by their exorbitant cost, extremely high mass density, high toxicity of fluorinated salts, and exceptionally sluggish ionic conductivity at room temperature caused by high dynamic viscosity. To circumvent these critical bottlenecks, the advanced concept of Localized High-Concentration Electrolytes (LHCE) was introduced into the zinc battery domain [47]. LHCEs are strategically formulated by introducing an inert, non-solvating diluent—typically highly fluorinated ethers such as 1,1,2,2-tetrafluoroethyl-2,2,3,3-tetrafluoropropyl ether (TTE) or bis(2,2,2-trifluoroethyl) ether (BTFE)—into a primary WIS precursor.

The genius of the LHCE design lies in decoupling localized solvation chemistry from bulk fluid dynamics. LHCEs are formulated by introducing an inert, non-solvating diluent—typically highly fluorinated ethers possessing near-zero donicity (donor number ≈ 0)—into a primary WIS precursor. Because the highly electronegative diluent does not participate in the primary coordination of Zn^2+^, it perfectly preserves the localized anion-rich primary solvation structure (CIPs and AGGs) established by the concentrated salts, maintaining the superior SEI-forming and anti-shuttling properties of the WIS [48]. Simultaneously, the non-polar diluent thoroughly breaks the macroscopic viscosity of the system, successfully restoring fluid dynamics and enhancing bulk ionic conductivity by an order of magnitude. Crucially, from an industrial standpoint, the diluent expands the fluid volume, which drastically reduces the overall consumption of expensive and heavy active salts, thereby elegantly resolving the economic and mass density constraints while maintaining rigorous interfacial protection [49,50].

Despite the profound thermodynamic and interfacial benefits, a critical evaluation of the Water-in-Salt (WIS) strategy reveals severe trade-offs that currently impede its commercial viability. The fundamental reliance on massive salt concentrations inherently results in exceptionally high dynamic viscosity. This physically restricts overall ion mobility, leading to significantly reduced Zn^2+^ transference numbers and exceptionally poor low-temperature transport kinetics. Consequently, purely WIS-based AZIBs often suffer from severe concentration polarization under high-rate operation. Furthermore, from a practical and scalable perspective, the overarching reliance on massive quantities of highly fluorinated salts (such as Zn(TFSI)_2_) introduces prohibitive economic costs and raises serious environmental and toxicity concerns regarding their synthesis, widespread deployment, and end-of-life recycling. Therefore, while WIS serves as a powerful proof-of-concept for thermodynamic regulation, its universal application is strictly bottlenecked by these macroscopic physical and economic realities.

### 3.2. Cosolvent Electrolyte Engineering: Tuning the Hydrogen-Bonding Network

While WIS and LHCE strategies heavily rely on manipulating the anionic coordination chemistry via massive salt concentrations, cosolvent engineering offers a more economically viable, structurally tunable, and diverse approach. This strategy focuses on rationally manipulating the hydrogen-bonding network and the dielectric properties of the bulk electrolyte through the introduction of specific miscible organic solvents [51]. By blending water with carefully selected organic solvents—such as propylene carbonate (PC), ethylene glycol (EG), dimethyl sulfoxide (DMSO), acetonitrile (AN), or various primary and secondary alcohols—researchers can precisely tune the physicochemical parameters of the electrolyte to simultaneously satisfy the conflicting demands of the cathode and anode [52,53].

The fundamental mechanism of cosolvent engineering revolves around the concept of the Donor Number (DN) and Gutmann’s Acceptor Number (AN). When a strong electron-donating organic cosolvent (for example, DMSO, which possesses a significantly higher DN than water) is introduced into the aqueous system, the highly electronegative oxygen atoms of the sulfoxide group form much stronger dipole–cation interactions with the Zn^2+^ ions than the oxygen atoms in H_2_O. Consequently, guided by thermodynamic stability, the organic molecules preferentially enter and dominate the primary solvation sheath of Zn^2+^ [54]. This molecular substitution process physically expels H_2_O molecules from the inner coordination sphere. During the charging process, as the solvated Zn^2+^ complex approaches the highly polarizing electric field of the inner Helmholtz plane at the anode, the easily reducible water molecules are safely shielded in the outer secondary solvation sheath or the bulk fluid, effectively preventing them from undergoing reductive decomposition and triggering HER [55,56].

Furthermore, from a macroscopic fluid dynamics perspective, the organic cosolvents serve as powerful “hydrogen bond disruptors.” The intricate addition of solvents like EG or glycerol introduces robust intermolecular hydrogen bonds between the organic hydroxyl groups and the aqueous protons. This interaction systematically fragments the continuous, long-range three-dimensional hydrogen-bonding network of bulk water into isolated, localized clusters [57]. This fragmentation critically restricts the rapid, free migration of active water molecules toward the metallic zinc anode surface, thereby thoroughly stifling the continuous parasitic corrosion and the subsequent formation of insulating byproducts like zinc hydroxide sulfate.

A fascinating and highly advanced kinetic compensation mechanism has also been recently proposed and validated for specific cosolvent systems [58]. Although introducing viscous organic solvents typically lowers the overall macroscopic ionic conductivity of the electrolyte, utilizing cosolvents with specific dielectric constants can actually increase the critical ion pair distance within the bulk fluid. This widening significantly weakens the electrostatic interaction between the Zn^2+^ cations and the surrounding counter-anions (like SO_4_^2−^ or CF_3_SO_3_^−^). This electrostatic decoupling elevates the Zn^2+^ transference number (t_Zn2+_), compensating for the bulk viscosity drag and ensuring that rapid, unimpeded desolvation kinetics can occur at the solid–liquid interface, enabling exceptional high-rate performance [59].

On the iodine cathode side, the organic/water hybrid environment drastically alters the polarity and solvation capability of the fluid. The mixed solvent system essentially acts as a precisely tuned anti-solvent for the highly polar polyiodide species. By lowering the macroscopic dielectric constant of the electrolyte, the cosolvent increases the thermodynamic energy barrier for I_3_^−^ dissolution. The multi-phase I_2_/I^−^ conversion reaction is thereby strictly confined within the nanopores of the cathode carbon matrix. For instance, the strategic introduction of a TEP (triethyl phosphate) cosolvent has been demonstrated to almost entirely eradicate the presence of free I_3_^−^ in the electrolyte, extending the cycle life of the AZIB to thousands of hours under high depth-of-discharge (DOD) conditions, while virtually eliminating the self-discharge phenomenon that plagues purely aqueous systems [60,61].

### 3.3. Functional Electrolyte Additives: The “Less Is More” Paradigm

While massive concentrations of salts or large volume fractions of cosolvents can fundamentally alter the bulk fluid properties, the most cost-effective, easily scalable, and operationally simple strategy in electrolyte engineering is the strategic introduction of functional additives [62]. Unlike cosolvents that constitute a major percentage of the liquid, additives are typically utilized in minuscule trace amounts—often at the parts-per-million (ppm) level—or ranging strictly from 0.1 wt.% to 5 wt.%. The “less is more” philosophy relies on leveraging the unique molecular architectures, functional groups, and extreme adsorption energies of these additives to aggressively target and stabilize the specific electrode/electrolyte interfaces, without perturbing the high ionic conductivity of the bulk aqueous medium [63]. Additives for AZIBs are generally classified into three mechanistic categories: cationic, anionic, and highly functionalized organic molecules.

#### 3.3.1. Cationic Additives for Electrostatic Shielding

Large, electrochemically inert organic or inorganic cations—such as tetrabutylammonium (TBA^+^, cetyltrimethylammonium (CTA^+^), or even trace amounts of Na^+^ and K^+^-function primarily via the classic and elegant “electrostatic shield” mechanism [64]. The efficacy of this strategy relies on a strict thermodynamic prerequisite: the reduction potentials of these additive cations must be significantly lower (more negative) than the standard redox potential of Zn^2+^/Zn. Consequently, during the electrodeposition process, these cations remain electrochemically stable and do not undergo reduction or co-deposition. Instead, driven by the intense applied electric field, they rapidly migrate toward the anode. Due to the tip-effect, microscopic protrusions and emerging dendrite nuclei inherently possess highly concentrated local electric fields. The inert cations preferentially adsorb onto these high-energy “hotspots”. Their localized accumulation establishes a highly concentrated, positively charged electrostatic barrier exactly where a dendrite would normally propagate. This localized positive charge strongly repels the incoming flux of similarly charged Zn^2+^ ions, forcing the active zinc species to detour and deposit in the adjacent, thermodynamically less favorable “valley” regions [65]. By continuously redirecting the ionic flux from the peaks to the valleys, this dynamic, self-healing shielding mechanism enforces a rigorously smooth, highly dense, and dendrite-free zinc morphology over extended cycling.

#### 3.3.2. Anionic and Salt Additives for Thermodynamic Pre-Saturation

A uniquely brilliant strategy specific to AZIBs involves manipulating the common ion effect and complexation equilibria using anionic additives [66]. For example, explicitly utilizing ZnI_2_ salt as a concentrated additive (or directly as the primary electrolyte salt) serves a profound dual purpose. Firstly, introducing a massive excess of I^−^ ions into the bulk electrolyte forces the dissolution equilibrium (I_2_ + I^−^ ⇌ I_3_^−^) to shift drastically, effectively pre-saturating the electrolyte and severely suppressing the further outward dissolution of active iodine from the cathode. Secondly, this immense iodide reservoir provides an extra active material inventory, directly participating in the energy storage process and unlocking ultra-high areal capacities. Furthermore, specific halide anions (like Cl^−^ or Br^−^) can be introduced to strongly adsorb on the zinc surface, expelling water molecules from the inner Helmholtz layer and guiding the preferential exposure of the highly stable, low-surface-energy Zn (002) facets, thereby mitigating localized corrosion [67,68].

#### 3.3.3. Dual-Function Molecular Additives and Multi-Electron Catalysis

The absolute cutting-edge of AZIB electrolyte research is currently dominated by the design of dual-function molecular additives. These are typically complex organic molecules featuring rich heteroatoms (N, O, S, P) and highly conjugated π-electron systems—such as specific amino acids (e.g., L-lysine), cyclic oligosaccharides (e.g., cyclodextrins), crown ethers, or biomolecules like caffeine and lactulose [69,70]. These molecules act as bilateral interfacial stabilizers.

On the anode side, the strongly polar functional groups (like the carbonyl or amine groups) dynamically coordinate with the Zn^2+^ ions, partially replacing water in the primary solvation sheath. Upon reaching the interface, these molecules robustly chemisorb onto specific crystallographic planes of the zinc substrate, displacing active water and creating a hydrophobic interfacial layer that utterly stifles HER. Simultaneously, on the cathode side, the conjugated electron-rich structures act as incredibly powerful chemical anchors. Through synergistic strong Lewis acid–base interactions, van der Waals forces, and hydrogen bonding, they firmly capture and immobilize the highly electrophilic I_3_^−^ and I_5_^−^ intermediate species [71,72].

Most groundbreaking of all, these molecular additives are the key to unlocking the elusive multi-electron transfer mechanisms in AZIBs. Traditional AZIBs operate strictly on a two-electron (2I^−^/I_2_) conversion process. However, specific complexing agents, particularly those based on quaternary ammonium compounds or sophisticated dual-function molecules like hexamethylenetetramine (HMTA), can fundamentally alter the reaction thermodynamics [73]. By encapsulating the highly reactive, positively charged iodine cation (I^+^) within a protective coordination pocket, these additives prevent its violent, spontaneous hydrolysis by water. This incredible stabilization enables a highly reversible four-electron redox cycle (2I^−^ ⇌ I_2_ ⇌ 2I^+^). This extraordinary leap not only doubles the theoretical specific capacity but also significantly elevates the discharge voltage plateau, drastically amplifying the ultimate energy density ceilings of AZIBs and opening an entirely new era for halogen-based electrochemistry [74,75].

While the four-electron (2I^−^ ⇌ I_2_ ⇌ 2I^+^) conversion promises extraordinary specific capacities, it is crucial to recognize that the stabilization of I^+^ is not a generally accepted or easily achievable phenomenon in aqueous media. The I^+^ cation is intrinsically highly electrophilic and thermodynamically unstable in the presence of free water. Without profound microenvironmental protection, I^+^ undergoes spontaneous and rapid hydrolysis to form hypoiodous acid (HOI), following the pathway: I^+^ + H_2_O → HOI + H^+^. This intermediate is highly unstable and subsequently disproportionates into iodate (IO_3_^−^) and iodide (I^−^) via 3HOI → IO_3_^−^ + 2I^−^ + 3H^+^. Because the electrochemical reduction of IO_3_^−^ is kinetically sluggish and highly irreversible under typical battery conditions, this hydrolysis pathway represents a catastrophic and permanent loss of active material. Therefore, successfully achieving the four-electron process rigorously requires sophisticated electrolyte engineering—such as utilizing complexing agents (e.g., specific halides like Cl^−^ and Br^−^, or strong Lewis basic quaternary ammoniums)—to heavily coordinate the I^+^ center, lowering its LUMO energy, and thoroughly shielding it from nucleophilic attack by bulk water molecules.

#### 3.3.4. Deep Eutectic Solvents (DES): Engineering a Water-Free Network

In the relentless pursuit of entirely circumventing the intrinsic detriments of free water molecules, Deep Eutectic Solvents (DES) have emerged as revolutionary, ultra-low-cost, inherently safe, and environmentally benign alternatives to traditional room temperature ionic liquids [76]. A typical DES is elegantly synthesized by gently mixing a solid metal salt (acting as the hydrogen bond acceptor, HBA, such as hydrated zinc chloride, ZnCl_2_, or zinc nitrate hexahydrate) with a solid organic hydrogen bond donor (HBD, such as urea, acetamide, or ethylene glycol) at specific stoichiometric molar ratios [77]. Upon mixing and mild heating, the immense, chaotic hydrogen-bonding network newly established between the HBA and HBD profoundly depresses the lattice energy of the original solid precursors, resulting in a stable, highly viscous liquid state at room temperature [78].

In a pristine DES electrolyte system, the concept of “bulk free water” is virtually obliterated. All residual water molecules—which strictly originate from the hydration spheres of the metal salts—are relentlessly trapped and geometrically confined within the extensive, rigid hydrogen-bonded framework formed by the HBDs [79]. This profound atomistic confinement entirely eradicates the thermodynamic possibility of parasitic water reduction (HER) and inherently prevents the formation of massive insulating byproducts like basic zinc sulfates.

**Figure 3:**
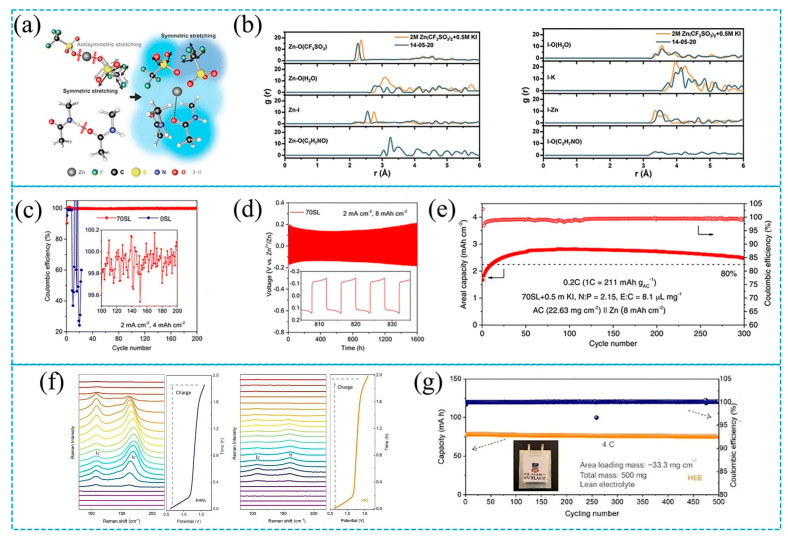
(**a**) Schematic representation of eutectic solution formation mechanism with varying Zn(CF_3_SO_3_)_2_/N-ACE molar ratios from 1:2 to 1:8. (**b**) Calculated radial distribution functions (RDFs) for Zn^2+^ and I^−^ from MD simulations in 2 M Zn(CF_3_SO_3_)_2_ + 0.5 M KI and 14-05-20 solutions, respectively. (**c**) CE of Zn||Cu batteries at 2 mA cm^−2^ with a capacity of 4 mA h cm^−2^. (**d**) Galvanostatic Zn stripping/plating observed in Zn||Zn symmetric batteries with 70SL at 2 mA cm^−2^ with a capacity of 8 mA h cm^−2^. (**e**) Long-term cycling performance of high-loading AC in 70SL + 0.5 m KI at 0.2C with a controlled N/P ratio of 2.15. (**f**) In situ Raman spectroscopy used to monitor the changes on the electrode surface in aqueous ZnSO_4_ or HEE. (**g**) Cycling longevity of Zn-I_2_ pouch cell tested at 4C, with the inset depicting the pouch cell designed for high mass loading of 33.3 mg cm^−2^ [58].

For the AZIB system, the DES provides a truly unprecedented coordination environment that acts as a fortress against degradation. The extraordinarily abundant functional groups (e.g., the dense amide groups in a urea-based DES, or the massive hydroxyl networks in an EG-based DES) form exceptionally dense, interlocking complexation networks with the dissolved iodine species [80]. This immense structural rigidity and high localized viscosity severely limit the diffusion coefficient of the I_3_^−^ anions. The polyiodides are thereby securely trapped at the cathode interface, completely suffocating the shuttle effect, while the system still supports surprisingly exceptional plating and stripping reversibility at the Zn anode (Figure 3).

However, pure DES systems often suffer from lethargic reaction kinetics and high ohmic polarization due to their massive viscosity. To resolve this, recent breakthroughs have introduced the ingenious “Water-in-DES” concept. By precisely titrating specific volumes of water back into the pure DES, researchers can strike a masterful, delicate balance: dramatically enhancing the ionic conductivity to rival traditional aqueous systems, while maintaining the robust stabilizing capabilities, the widened voltage window, and the anti-shuttling prowess of the deep eutectic network [81].

While Deep Eutectic Solvents (DESs) offer an unparalleled, water-free environment for polyiodide confinement, it is crucial to critically acknowledge their inherent operational trade-offs. The intense, extensive hydrogen-bonding network that successfully traps iodine species simultaneously establishes an environment of extreme macroscopic viscosity. This intrinsic characteristic leads to fundamentally sluggish multiphase conversion kinetics and significantly increased ohmic and charge transfer polarization compared to standard aqueous systems. Most critically, the high viscosity and distinct surface tension of pure DESs severely compromise their physical compatibility with practical cell designs. They exhibit exceptionally poor penetrability and wetting capabilities when applied to thick, commercial-grade porous electrodes. As a result, the impressive cyclic stability of DES-based AZIBs is frequently demonstrated only at relatively low areal capacities and thin electrode loadings, highlighting a significant gap between fundamental interfacial stabilization and practical, high-energy-density scale-up.

While strategies such as Water-in-Salt (WIS) and Deep Eutectic Solvents (DES) are highly celebrated for thermodynamically suffocating the polyiodide shuttle effect via mass transport confinement, a critical kinetic perspective must be emphasized. The inherently high viscosity and strong solute–solvent coordination within these engineered electrolytes dramatically increase the activation energy required for the solid–liquid I_2_ ⇌ I^−^ conversion. Consequently, the interfacial electron transfer mechanisms become severely restricted, leading to high charge transfer resistance (R_ct)_ and sluggish oxidation/reduction kinetics. To circumvent this kinetic penalty, merely trapping the polyiodides is insufficient; the cathode interface must be electrocatalytically active. Advanced strategies now emphasize integrating heteroatom-doped carbon matrices or transition metal single-atom catalysts to dynamically interact with the I_3_^−^/I_5_^−^ intermediates. These electrocatalytic sites facilitate rapid orbital hybridization, lower the energy barrier for the breaking and formation of I-I bonds, and thereby ensure that the multielectron transfer processes proceed with minimal overpotential, balancing spatial confinement with accelerated reaction kinetics.

Furthermore, from a macroscopic perspective, the introduction of viscous cosolvents or highly concentrated salts fundamentally alters the fluid dynamics of the electrolyte. The increased molecular friction and the formation of bulkier solvation sheaths severely depress the diffusion coefficients (D) of the active iodine species. Consequently, at high current densities, the rate-determining step shifts dramatically from interfacial charge transfer to bulk mass transport. Most critically, the extreme viscosity associated with these advanced electrolytes directly compromises cathode utilization. Highly viscous fluids struggle to deeply penetrate the tortuous micro- and mesopores of practical, high-loading carbon hosts. This inherently poor interfacial wetting leads to the formation of electrochemically “dead zones” within the cathode interior, locking away active materials and significantly curtailing the realizable areal capacity of the battery.

#### 3.3.5. Quasi-Solid-State and Hydrogel Electrolytes for Extreme Environments

**Figure 4:**
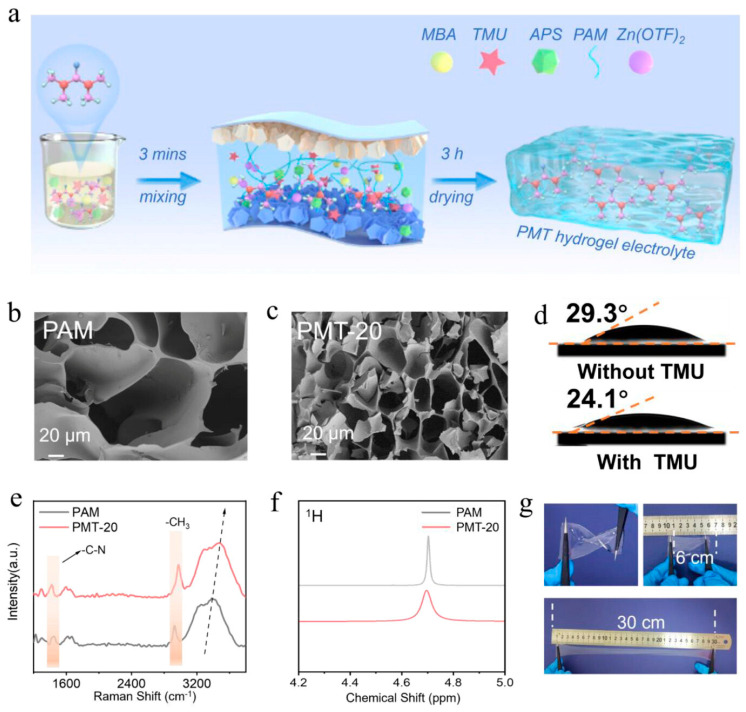
(**a**) Schematic diagram of PMT hydrogel electrolyte synthesis. SEM image of (**b**) the PAM electrolyte and (**c**) the PMT-20 electrolyte. (**d**) Contact angles between PAM and Zn (up), PMT-20 and Zn (down). (**e**) The Raman spectra of the PAM and PMT-20 electrolytes. (**f**) 1H NMR spectra of PAM and PMT-20 electrolytes. (**g**) Pictures of the twisted, original, and stretched PMT hydrogel electrolyte [24].

As the global technological landscape rapidly shifts toward flexible, wearable, and inherently safe electronic architectures—such as smart textiles, implantable medical devices, and foldable displays—replacing highly fluid, leak-prone liquid electrolytes with quasi-solid-state or hydrogel electrolytes has become an absolutely critical trajectory for the evolution of AZIBs [82]. Hydrogel electrolytes are sophisticated composite materials, formulated by intricately cross-linking hydrophilic polymer chains (such as polyacrylamide (PAM), polyvinyl alcohol (PVA), gelatin, or naturally derived nanocellulose) and subsequently swelling this massive 3D matrix with an aqueous zinc salt solution. This creates a unique hierarchical architecture that acts simultaneously as both the electrolyte and the physical separator (Figure 4).

The immense superiority of hydrogel electrolytes in mitigating AZIB failure modes stems from an exquisite combination of profound physical and chemical confinements. Physically, the tortuous, nanometer-scale pores of the interconnected polymer network act as a formidable steric barrier. The sheer size of the intricate polymer meshes significantly impedes the rapid diffusion of the bulky, long-chain I_3_^−^ and I_5_^−^ anions, while still allowing the relatively unimpeded, rapid transport of the smaller, partially desolvated Zn^2+^ cations. Chemically, the polymer backbones are meticulously engineered to be decorated with a dense array of polar functional groups (-OH, -NH_2_, -COOH, -SO_3_^−^). This functionalized matrix acts as a macroscopic array of chemical traps, tightly binding the electrophilic polyiodides via intense hydrogen bonding and dipole–dipole interactions, thereby thoroughly and permanently mitigating the shuttle effect at its source.

Furthermore, the implementation of hydrogel electrolytes endows AZIBs with exceptional environmental adaptability, solving the critical issue of extreme-temperature operation. By strategically introducing advanced antifreeze agents (such as glycerol, ethylene glycol, or highly concentrated chaotropic salts) into the polymer matrix, the intricate hydrogen bonds between the polymer chains and the encapsulated water molecules are radically restructured. This profound disruption drastically depresses the freezing point of the confined water, completely preventing the formation of rigid, ion-blocking, and structurally destructive ice crystals at sub-zero temperatures. Consequently, this enables robust, high-capacity AZIB operation in extreme cold environments (e.g., reaching down to 40 °C or even 60 °C), expanding the geographic deployment potential of these batteries.

Additionally, the intrinsic mechanical toughness, stretchability, and self-healing properties of specific dynamic covalent hydrogels are paramount for modern applications. Utilizing reversible bonds (such as boronate ester bonds, hydrogen bonds, or metal–ligand coordination), the hydrogel can autonomously repair mechanical fractures. This allows the fully assembled AZIB devices to withstand severe, repeated mechanical deformations—such as extreme bending, forceful twisting, heavy compression, or even direct severing—without suffering from catastrophic internal short circuits or hazardous electrolyte leakage, solidifying hydrogel-based AZIBs as the ultimate candidate for the next generation of safe, wearable energy storage technologies.

Despite their undisputed superiority in mitigating electrolyte leakage and physically suffocating the shuttle effect, hydrogel electrolytes introduce a fundamental confinement versus transport paradox that must be critically evaluated. The highly tortuous, densely cross-linked polymer networks that effectively restrict bulky polyiodide diffusion inevitably impose severe mass transport limitations on the active Zn^2+^ and I^−^ ions. This tortuosity inherently lowers the bulk ionic conductivity compared to free-flowing liquid electrolytes, often resulting in notable capacity decay at high current densities. Furthermore, the quasi-solid nature of the hydrogel creates formidable interfacial challenges. Achieving conformal, continuous, and dynamic physical contact—or wetting—between the rigid polymer matrix and the microscopic crevices of thick, porous carbon cathodes is notoriously difficult. This imperfect solid–solid or quasi-solid interfacial contact can lead to significant localized dead zones within the cathode, ultimately limiting the realization of maximum active material utilization in practical pouch cell configurations.

#### 3.3.6. Synergy Between Electrolyte and Separator Engineering

**Figure 5:**
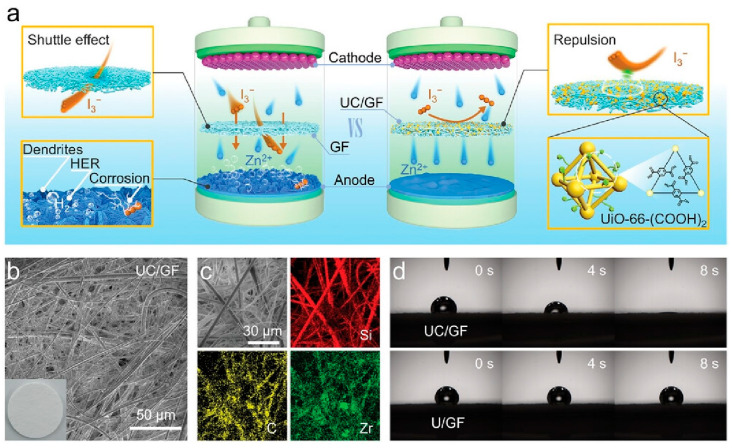
(**a**) Schematic illustration of the AZIBs assembled with commercial GF and UC/GF separators. (**b**) Optical photo (diameter = 16 mm) and the top view SEM image of the UC/GF separator. (**c**) Top view SEM image of the UC/GF separator after tearing its surface and the corresponding elemental mapping images of Si, C, and Zr. (**d**) Wettability tests for UC/GF and U/GF separators [52].

While electrolyte engineering provides the fundamental thermodynamic foundation for halting iodine dissolution, a holistic approach to mitigating the polyiodide shuttle effect must critically incorporate the synergistic role of macroscopic separator engineering (Figure 5). In highly demanding practical scenarios, liquid-phase thermodynamic confinement must be coupled with physical and electrostatic barriers to establish a robust second line of defense. Standard glass fiber or porous polyolefin separators possess massive macroscopic pores that offer zero resistance to the migration of dissolved polyiodides. To overcome this, advanced ion-selective membranes, such as Nafion-based or sulfonate-functionalized separators, are strategically deployed. These membranes operate on the principle of Donnan exclusion; their densely packed, negatively charged functional groups exert intense electrostatic repulsion against migrating polyiodide anions (I_3_^−^, I_5_^−^), strictly confining them to the cathode compartment while efficiently facilitating the transport of positively charged Zn^2+^ cations. Furthermore, precisely engineered crystalline porous materials—such as Metal–Organic Frameworks (MOFs) or Covalent Organic Frameworks (COFs)—can be coated onto the separator to provide unparalleled steric hindrance. The highly ordered sub-nanometer channels function as precise ionic sieves, physically blocking the bulky polyiodide complexes while allowing the smaller Zn^2+^ ions to traverse freely.

Beyond passive physical and electrostatic blocking, modern separator engineering is increasingly transitioning toward active chemical interception. By modifying the separator interface with highly polar carbonaceous networks, heteroatom-doped matrices, or transition metal catalysts, the separator actively captures escaping polyiodides via strong chemisorption. Crucially, these catalytically active interfaces can dynamically accelerate the redox conversion of the trapped polyiodides, chemically recovering the active material inventory. Therefore, the ultimate realization of highly reversible AZIBs inherently depends on the seamless co-design of both the electrolyte’s solvation chemistry and the separator’s permselective architecture.

## 4. Advanced Characterizations and Theoretical Computations

The rapid evolution of electrolyte engineering in AZIBs has comprehensively transitioned from traditional, empirical trial-and-error formulations to rational, predictable, and molecular-level design. This profound paradigm shift is fundamentally driven by the development and deep integration of advanced in situ/operando characterization techniques coupled with multiscale theoretical computations [83]. Traditional ex situ characterizations, which involve disassembling the battery to analyze the electrodes, often fail catastrophically to capture the transient, highly dynamic, and metastable states of the active chemical species. The washing and drying processes inevitably destroy the fragile solid–electrolyte interphase (SEI) and permanently alter the solvation environments. Therefore, cutting-edge research relies almost exclusively on operando techniques and quantum chemical simulations to bridge macroscopic electrochemical performance with microscopic atomic dynamics, providing indisputable evidence for the proposed stabilization mechanisms.

### 4.1. Operando Spectroscopic and Dynamic Interfacial Tracking

To fully comprehend the success of any advanced electrolyte engineering strategy, researchers must continuously monitor the structural and chemical evolution of both the bulk electrolyte and the electrode/electrolyte interfaces under realistic electrochemical polarization scenarios.

#### 4.1.1. Multidimensional Vibrational and Magnetic Resonance Spectroscopy

To accurately decipher the multiphase solid–liquid–solid conversion unique to AZIBs, advanced optical spectroscopies are absolutely indispensable. While generic battery studies rely on these tools for solvent analysis, AZIB research demands their application for rigorous iodine speciation. Operando UV–Vis spectroscopy stands as the ultimate quantitative technique for monitoring the polyiodide shuttle effect in real time. Because triiodide (I_3_^−^) exhibits highly intense and distinct molar absorptivity peaks at approximately 288 nm and 353 nm, operando UV–Vis directly visualizes the concentration gradient of dissolved active species traversing the separator. A perfectly engineered electrolyte will suppress these absorption bands to near baseline levels during cycling. Complementarily, operando Raman spectroscopy is vital for tracking the transient solid/liquid speciation at the cathode interface. The distinctive symmetric stretching vibrations of linear I_3_^−^ (at ~110–115 cm^−1^) and the V-shaped I_5_^−^ complex (at ~160–170 cm^−1^) provide an undeniable spectral fingerprint, revealing precisely when and how the I_2_ solid phase is consumed and whether intermediate polyiodides are successfully confined or irreversibly dissolved [84].

In situ Raman spectroscopy has established itself as an absolutely indispensable tool for tracking the intricate, multi-phase solid–liquid–solid conversion of iodine species in real time. Because elemental iodine molecules and the various polyiodide anions possess distinctly different polarizabilities and symmetry geometries, they exhibit highly specific and quantifiable Raman scattering shifts. During a standard discharge process, the gradual disappearance of the characteristic solid I_2_ peak and the subsequent emergence of intense, distinct bands located around 110–115 cm^−1^ (assigned unequivocally to the symmetric stretching vibration of the linear I_3_^−^ anion) and 160–170 cm^−1^ (assigned to the V-shaped I_5_^−^ complex) explicitly reveal the transient conversion pathways [84,85]. When highly advanced electrolyte strategies—such as the introduction of molecular electrocatalysts, deep eutectic solvents, or strong Lewis basic cosolvents—are applied, operando Raman mapping conclusively demonstrates a drastic reduction in the measurable lifetime, concentration, and signal intensity of the I_3_^−^ intermediates. This spectroscopic evidence definitively proves that the electrolyte modification successfully accelerates the conversion kinetics and effectively locks the active species within the cathode matrix, thereby thoroughly suppressing the shuttle effect [86].

Complementary to Raman analysis, in situ Fourier-Transform Infrared (FTIR) spectroscopy is heavily utilized to deeply probe the dynamic molecular fluctuations of the ubiquitous hydrogen-bonding network within the aqueous electrolyte. The precise position, symmetry, and full-width at half-maximum (FWHM) of the broad O-H stretching vibration band (typically spanning from 3200 to 3600 cm^−1^) directly correlate with the local hydrogen bond strength. A significant spectral blue-shift (moving toward higher wavenumbers) combined with a narrowing of this band serves as direct, irrefutable evidence that functional additives, massive salt concentrations, or organic diluents have successfully disrupted the extended, long-range bulk water structure, effectively lowering water activity and mitigating the parasitic hydrogen evolution reaction (HER) [87].

Furthermore, advanced Nuclear Magnetic Resonance (NMR) spectroscopy has become critical for deciphering the precise solvation structure. While optical spectroscopy provides vibrational data, Liquid-state ^1^H NMR and ^17^O NMR precisely monitor the chemical shifts associated with the proton and oxygen nuclei of water. When water molecules are displaced from the intensely polarizing primary solvation sheath of Zn^2+^ by specific engineered anions (like trifluoromethanesulfonate, OTf^−^) or organic ligands, the electron cloud density around the water nuclei changes profoundly, resulting in quantifiable upfield or downfield chemical shifts. Additionally, solid-state NMR (ssNMR), particularly ^19^F and ^13^C cross-polarization magic-angle spinning (CP-MAS) techniques, is paramount for analyzing the exact chemical composition and structural connectivity of the organic–inorganic composite SEI layer that forms at the zinc anode interface during cycling.

#### 4.1.2. High-Resolution Morphological and Mass-Gravimetric Analysis

While spectroscopy details molecular bonds, understanding the physical degradation of the zinc anode requires sophisticated morphological tracking. In situ optical microscopy allows researchers to visually monitor the highly aggressive, localized growth of zinc dendrites in real-time across the cross-section of a transparent capillary cell. Unregulated dilute electrolytes rapidly exhibit dark, needle-like protrusions that violently short-circuit the cell. Conversely, successfully engineered electrolytes demonstrably guide smooth, bottom-up planar deposition, completely eliminating the notorious “tip-effect” [88].

To push the resolution to the atomic limit, Cryogenic Electron Microscopy (Cryo-EM) has recently revolutionized the study of AZIB interfaces [89]. Traditional transmission electron microscopy (TEM) uses an intense electron beam that instantly melts and destroys the highly volatile, water-rich, and fragile SEI components. Cryo-EM circumvents this by flash-freezing the electrode at liquid nitrogen temperatures, preserving the native, solvated state of the interfacial layers. This allows researchers to definitively observe the nanoscale thickness, the exact crystalline domain distribution (such as nanometer-sized ZnF_2_ or ZnS precipitates embedded in an amorphous polymer matrix), and the specific lattice fringes of the epitaxially deposited zinc metal.

Concurrently, Electrochemical Quartz Crystal Microbalance (EQCM) with dissipation monitoring provides an extraordinary, real-time mass-gravimetric perspective. By measuring nanogram-level mass changes simultaneously with the applied electrochemical current, EQCM precisely calculates the apparent molecular weight (M_w_) of the depositing or dissolving species. This technique directly answers whether a pure, desolvated Zn^2+^ ion (M_w_ = 65.38 g/mol) is depositing, or if massive, hydrated coordination complexes and parasitic byproducts (like basic zinc sulfate) are co-precipitating onto the anode surface, providing undeniable kinetic proof of the desolvation efficiency managed by the electrolyte [90].

While Electrochemical Quartz Crystal Microbalance (EQCM) is frequently utilized to study Zn^2+^ desolvation at the anode, its application at the iodine cathode provides profound, AZIB-specific gravimetric insights. By continuously monitoring the resonant frequency shift of a coated quartz crystal during galvanostatic cycling, cathode-focused EQCM computes the exact mass-per-electron (mpe) ratio of the redox events. This technique explicitly distinguishes the physical state of the active materials: it quantitatively separates the mass increase corresponding to solid I_2_ precipitation from the rapid mass loss associated with detrimental polyiodide dissolution into the bulk liquid. Furthermore, EQCM exquisitely captures complex transient phenomena, such as the formation of dense liquid–polyiodide coacervate phases at the electrode–electrolyte interface, offering a definitive kinetic map of how electrolyte engineering confines mass transport directly at the cathode boundary [90].

#### 4.1.3. Synchrotron Radiation X-Ray Techniques

For the most definitive, atomistic resolution of both solid-state crystallography and liquid-state coordination environments, synchrotron radiation-based techniques are deployed. In situ X-ray Diffraction (XRD), utilizing high-intensity synchrotron beams, continuously tracks the crystallographic evolution of the zinc anode. The real-time evolution of the integrated intensity ratio between the closely packed Zn (002) plane and other planes (like the (101) facet) serves as a quantitative indicator of texture evolution. A continuously dominant (002) diffraction signal guarantees that the electrolyte dynamically forces incoming zinc ions to adopt a thermodynamically stable, horizontal growth mode, intrinsically preventing vertical dendrite penetration [89].

Moreover, X-ray Absorption Spectroscopy (XAS) is universally acknowledged as the gold standard for defining liquid-state solvation sheaths [91]. Because XAS is completely unaffected by the long-range disorder of liquid systems, it is perfectly suited for scrutinizing the central Zn^2+^ ion. The X-ray Absorption Near-Edge Structure (XANES) region sensitively reports the localized oxidation state and the specific geometry of the unoccupied electronic orbitals. Concurrently, the Extended X-ray Absorption Fine Structure (EXAFS) region is mathematically isolated and Fourier-transformed into Radial Structure Functions (RSFs). The RSF plots explicitly reveal the exact atomic distances and coordination numbers (CN) between the central Zn^2+^ ion and its nearest coordinating neighbors. For example, explicitly demonstrating the reduction of the Zn-O(water) coordination number from 6.0 in a standard electrolyte to approximately 2.1 in a high-concentration electrolyte, simultaneously accompanied by the appearance of a strong Zn-O(anion) scattering path, provides absolute, unassailable proof of primary solvation sheath reconstruction [92]. Additionally, Small-Angle X-ray Scattering (SAXS) is increasingly utilized to identify mesoscale heterogeneities, explicitly revealing the formation of unique micelle-like structural networks or extensive ionic aggregates (AGGs) specifically within localized high-concentration electrolytes (LHCE).

Beyond basic phase identification, the advent of multi-electron (e.g., I^−^/I^0^/I^+^) halogen chemistry necessitates exceptionally sensitive electronic and local-structure probes. High-resolution X-ray Photoelectron Spectroscopy (XPS), particularly the careful deconvolution of the I 3d core-level spectra, is the absolute gold standard for verifying the stabilization of the highly electrophilic iodine cation (I^+^). Observing a definitive binding energy shift to higher eV values (characteristic of the +1 oxidation state), distinct from standard I_2_ (0) and I^−^ (−1), provides the definitive proof required to validate the four-electron redox pathway. Furthermore, while conventional studies frequently employ Zn K-edge XAS to determine primary solvation sheaths, AZIBs specifically demand Iodine K-edge (or L_III_-edge) X-ray Absorption Spectroscopy (XAS). Analyzing the Iodine EXAFS region allows researchers to meticulously extract the local coordination numbers and bond distances surrounding the active iodine center. This is paramount for proving whether iodine is merely physically adsorbed within a carbon host, or if it is chemically anchored via strong Lewis acid–base interactions and halogen bonding orchestrated by specific electrolyte additives.

### 4.2. Multiscale Quantum and Thermodynamic Computations

While advanced experimental observations masterfully report the physical outcomes of electrochemical processes, they cannot inherently explain the fundamental driving forces at the sub-atomic level. To uncover the quantum mechanical origins and thermodynamic landscapes of these phenomena, multi-scale computational modeling—specifically Molecular Dynamics (MD) and Density Functional Theory (DFT)—is absolutely mandatory [93].

#### 4.2.1. Classical and Ab Initio Molecular Dynamics (MD/AIMD)

Classical Molecular Dynamics (MD) simulations are uniquely capable of bridging the vast gap between macroscopic physical fluid properties (such as viscosity, surface tension, and overall ionic conductivity) and the microscopic, atomic-level solvation structures. By precisely computing the complex trajectories of tens of thousands of atoms over nanosecond to microsecond timescales, researchers generate the Radial Distribution Function (RDF, denoted as g(r)). The RDF plots unequivocally and statistically define the probability of finding specific atoms (like the oxygen of a water molecule or the fluorine of an anion) at an exact radial distance from a central Zn^2+^ cation. By mathematically integrating the precise area under the first prominent RDF peak, researchers accurately calculate the exact dynamic coordination number, thereby quantitatively proving how effectively engineered additives or bulky salts have expelled active H_2_O molecules from the inner Helmholtz plane [94]. Furthermore, classical MD calculations extract the Mean Square Displacement (MSD), providing crucial theoretical values for the diffusion coefficients of active polyiodides, rigorously explaining the macroscopic viscosity and conductivity phenomena.

To achieve even deeper accuracy regarding bond breaking and formation, Ab Initio Molecular Dynamics (AIMD) is employed. Unlike classical MD, which relies on pre-defined empirical force fields, AIMD computes the forces dynamically “on the fly” directly from the electronic structure using quantum mechanical principles. AIMD simulations provide unparalleled insights into the transient, picosecond-scale desolvation dynamics of the Zn^2+^ ion as it violently traverses the highly charged electrical double layer (EDL) immediately prior to deposition [95].

#### 4.2.2. Density Functional Theory (DFT) and Quantum Chemical Mechanisms

Density Functional Theory (DFT) serves as the absolute, undisputed theoretical backbone for evaluating thermodynamic descriptors, charge transfer kinetics, and orbital interactions at the deepest quantum mechanical level. In AZIB electrolyte research, DFT is extensively utilized to rigorously calculate the exact binding energies (or thermodynamic adsorption energies, ΔE_ads_) between specific zinc crystallographic facets (typically the stable (002) plane versus the high-energy (101) plane) and various competing electrolyte molecules. A significantly more negative ΔE_ads_ value computed for an engineered organic additive compared to a free water molecule theoretically dictates that the additive will spontaneously outcompete water for the active metallic surface sites, thereby establishing a highly stable, hydrophobic, and anti-corrosive protective molecular layer [96].

Moreover, DFT is critically essential for elucidating the remarkably complex and transient interactions occurring at the porous iodine cathode. To theoretically explain the successful suppression of the polyiodide shuttle effect, computational chemists calculate the exact interaction energies and the corresponding distance matrices between the highly electrophilic I_3_^−^ and I_5_^−^ intermediates and the specific Lewis basic functional groups of the strategically engineered electrolyte cosolvents or solid additives. By deeply analyzing the sophisticated Charge Density Difference (CDD) contours and the precise partial Density of States (pDOS), researchers can visually and mathematically pinpoint the exact direction and magnitude of interfacial electron transfer (for instance, highlighting the intense donation of lone-pair electrons from a nitrogen-rich additive directly into the lowest unoccupied molecular orbitals, LUMO, of the iodine species) [97].

To push the analysis further, advanced tools such as the Crystal Orbital Hamilton Population (COHP) are employed to meticulously partition the bonding interactions into bonding and anti-bonding contributions. This highest-level quantum analysis conclusively confirms whether advanced dual-function electrolyte molecules form robust, permanent coordination bonds with the active iodine species via synergistic σ-donation and π-backbonding mechanisms. Furthermore, advanced DFT protocols regularly employ the Nudged Elastic Band (NEB) method to search for theoretical transition states (TS). The NEB method accurately maps the Minimum Energy Pathway (MEP) and calculates the precise thermodynamic activation energy barriers (E_a_) required for specific reactions, such as the initial desolvation step of Zn^2+^ or the specific atomic migration steps of surface adatoms [98]. By explicitly demonstrating that a specifically designed electrolyte lowers the activation barrier for the I_2_ to I^−^ conversion while simultaneously raising the barrier for water reduction, DFT computations provide the ultimate, irrefutable closure to the proposed macroscopic hypotheses [99,100]. Through the seamless and rigorous integration of these profound computational quantum insights with high-resolution, operando spectroscopic and morphological evidence, the ongoing development of AZIB electrolytes is rapidly, confidently, and decisively moving toward an era of precise, fully predictable molecular-level engineering [101].

## 5. Conclusions and Perspectives

The search for safe, cheap, and powerful energy storage has made AZIBs a major research topic. As shown in this review, the main problems—zinc dendrites, hydrogen gas formation (HER), and the polyiodide shuttle effect—come from the activity of free water and the nature of the liquid electrolyte. In the past, simple physical blocks on the electrodes were not enough to stop these linked issues. Therefore, changing the electrolyte itself is now a more effective and complete solution.

By changing how ions bind and interact, researchers can control the zinc shell and how iodine reacts. Methods like high-concentration electrolytes, deep eutectic solvents, mixed fluids, and gels show that fixing the water network helps both sides of the battery at once. Also, using real-time testing tools and computer models has changed this work from guessing to exact design.

To systematically evaluate the efficacy of the diverse electrolyte engineering strategies discussed herein, we have summarized the critical electrochemical and physical metrics of representative AZIB systems in Table 1. A comparative analysis of these parameters—specifically ionic conductivity, Zn^2+^ transference number, voltage window, and realizable areal capacity—reveals the inherent trade-offs within current technological paradigms. For instance, while ultra-high-concentration systems (WIS) and Deep Eutectic Solvents (DES) substantially expand the electrochemical voltage window and suppress the iodine shuttle effect (yielding > 99.5 Coulombic efficiency), they inherently suffer from depressed ionic conductivity (<5 mS cm^−1^) compared to standard dilute aqueous electrolytes (~50 mS cm^−1^). Conversely, advanced localized high-concentration electrolytes (LHCE) and specifically functionalized hydrogels demonstrate a promising compromise, balancing rapid mass transport with robust interfacial confinement across wide operating temperatures. This tabulated quantitative framework is designed to guide future research toward balancing fundamental thermodynamic stability with practical, scalable performance metrics.

However, AZIBs still face a gap between lab tests and real-world sales. To move these batteries to the market, future work should focus on several key areas. Standard AZIBs use a two-electron process, which limits their total energy. The next big step is using the four-electron (2I^−^ ⇌ I_2_ ⇌ 2I^+^) cycle. The I^+^ intermediate reacts quickly with water, which is a problem. New electrolytes must have strong structures to shield the I^+^ from water molecules. Making this cycle stable will greatly increase the energy of AZIBs and help them compete with lithium-ion batteries. There are too many possible chemical mixtures to test by hand. This makes research slow and expensive. Future research should use Artificial Intelligence (AI) and Machine Learning (ML). By feeding computer data and test results into AI, programs can quickly find the best new molecules and salt mixtures. This will speed up the search for cheap, safe, and stable fluids. Batteries often look great in small lab tests but fail when built in larger sizes. Researchers must use strict and standard testing rules. Tests should use very little extra zinc, small amounts of electrolyte, and thick iodine layers. Also, moving to large flexible pouch cells brings new problems like heat control and gas pressure. The electrolyte must stop gas buildup to keep the battery from swelling or bursting. AZIBs must work in tough weather as green energy spreads globally. Gels and mixed fluids need to work in very cold conditions, such as below 40 °C. Also, many salts used today are toxic or expensive. This does not fit the goal of green energy. Future research must focus on electrolytes that are cheap, non-toxic, and easy to source in large amounts.

In summary, the renaissance of AZIBs, driven by sophisticated electrolyte engineering, represents a monumental leap forward in the quest for safe, scalable, and sustainable energy storage. By continuing to bridge the gap between profound atomic-level solvation thermodynamics and rigorous macroscopic cell engineering, the realization of commercially viable, high-energy AZIBs is rapidly approaching, promising to fundamentally reshape the landscape of the global energy grid.

## Figures and Tables

**Table 1:** Summary of key electrochemical metrics for representative electrolyte engineering strategies in aqueous zinc–iodine batteries.

Electrolyte Strategy	Representative Composition	Ionic Cond. (σ, mS cm^−1^)	t_Zn2+_	Voltage Window (V)	Cycle Life (Cycles @ Rate)	Areal Capacity (mAh cm^−2^)	CE (%)	Temp. Range (°C)
Baseline Aqueous	2 M ZnSO_4_	~50	0.3–0.4	~1.5	300 (@ 1 A g^−1^)	<1.0	~95.0	25
WIS/High-Conc.	1 M Zn(TFSI)_2_ + 20 M LiTFSI	1–5	0.6–0.7	>2.5	2000 (@ 2 A g^−1^)	1.5–2.0	>99.5	25
LHCE	WIS + TTE diluent	~15	0.75	~2.5	3000 (@ 5 A g^−1^)	>2.5	>99.8	−20 to 60
Anionic Additive	2 M ZnSO_4_ + 1 M ZnI_2_	~45	0.45	~1.7	5000 (@ 10 A g^−1^)	5.0	~99.0	25
Cationic Additive	2 M ZnSO_4_ + 0.1 M TBA^+^	~48	0.50	~1.8	1500 (@ 2 A g^−1^)	1.2	99.2	25
DES	ZnCl_2_/Urea	0.5–2	~0.5	>2.0	4000 (@ 2 A g^−1^)	1.0–3.0	~99.0	25
Hydrogel/Solid	PAM/PVA + ZnSO_4_	10–25	0.5–0.6	~1.8	2000 (@ 1 A g^−1^)	1.0–2.0	>99.0	−20 to 50

## Data Availability

No new data were created or analyzed in this study. Data sharing is not applicable to this article..
